# A Blockchain Framework for Securing Connected and Autonomous Vehicles

**DOI:** 10.3390/s19143165

**Published:** 2019-07-18

**Authors:** Geetanjali Rathee, Ashutosh Sharma, Razi Iqbal, Moayad Aloqaily, Naveen Jaglan, Rajiv Kumar

**Affiliations:** 1 Department of Computer Science and Engineering, Jaypee University of Information Technology, Waknaghat, Solan 173234, India; 2 Department of Electronics and Communication, Jaypee University of Information Technology, Waknaghat, Solan 173234, India; 3 College of Computer Information Technology, American University in the Emirates, Dubai 503000, UAE; 4 Gnowit Inc., 308 Legget Drive, Suite 206, Ottawa, ON K2K 1Y6, Canada

**Keywords:** connected vehicles, internet of vehicles, security, IoT, blockchain, vehicular ad-hoc network

## Abstract

Recently, connected vehicles (CV) are becoming a promising research area leading to the concept of CV as a Service (CVaaS). With the increase of connected vehicles and an exponential growth in the field of online cab booking services, new requirements such as secure, seamless and robust information exchange among vehicles of vehicular networks are emerging. In this context, the original concept of vehicular networks is being transformed into a new concept known as connected and autonomous vehicles. Autonomous vehicular use yields a better experience and helps in reducing congestion by allowing current information to be obtained by the vehicles instantly. However, malicious users in the internet of vehicles may mislead the whole communication where intruders may compromise smart devices with the purpose of executing a malicious ploy. In order to prevent these issues, a blockchain technique is considered the best technique that provides secrecy and protection to the control system in real time conditions. In this paper, the issue of security in smart sensors of connected vehicles that can be compromised by expert intruders is addressed by proposing a blockchain framework. This study has further identified and validated the proposed mechanism based on various security criteria, such as fake requests of the user, compromise of smart devices, probabilistic authentication scenarios and alteration in stored user’s ratings. The results have been analyzed against some existing approach and validated with improved simulated results that offer 79% success rate over the above-mentioned issues.

## 1. Introduction

In order to increase their own comforts, human daily life routines have been partially or completed replaced by embedded or automated machines in all aspects of business. Embedded systems monitor their environmental surroundings and subsequently respond or control the situation without any human intervention [1]. Recently, with the development of wireless communications and the advancement in vehicular industry, vehicular ad-hoc networks (VANETs) have become a mature research area. A VANET consists of a group of stationary and moving vehicles connected via a wireless network. Until recent times, the main use of VANETs was to provide comfort and safety to drivers in a vehicular environment [2]. However, this view is changing the infrastructure towards intelligent transportation systems where vehicles are connected and communicate using smart devices. Connected and Autonomous Electric Vehicles (CAEVs) is the most emerging vehicular technology among them all [3].The optimum value of this destructive technology has been seen a big successful business model for auto makers [4] [Vehicles that may connect to the internet and provide improved data sharing in the form of risk data, sensory and localization data and environmental perception is known as the internet of vehicles (IoV) or connected autonomous vehicles (CAV) [5]. In addition, with the continuous increase in urban population and rapid expansion of cities, vehicle ownership has been increasing at an exponential rate. CAV has been considered as one of the essential applications of VANETs where vehicles are becoming smarter by having sensors, adapters and control units for monitoring and communicating with their surroundings. A potential area for the application of CAV that has witnessed an unprecedented growth is in online cab booking services. The use of CAV yields a better result in vehicle entertainment experiences and helps in reducing the congestion by allowing the current information to be obtained by the vehicles instantly. Recently, online cab facilities or ride sharing services have drastically changed the public transportation industry and have been widely accepted by the users to avail the services at any time [6]. Further, it reduces the overhead of money negotiation between driver and customer and allows the customers to book their ride on a phone by tracking driver availability through global positioning systems (GPS). In spite of several advantages of using these services, there are various issues that need to be tackled, such as any person may register its vehicle number and provide online cab sharing services with the means of benefitting his/her personnel concerns by compromising the smart/IoT (internet of things) sensors/devices. However, until now, there exists no tracking systems or recording mechanisms that keep a check on compromised sensors or misbehavior with the customers during the rides, especially at night. A group of technical experts may forge the network system and increase or decrease their service ratings in order to continue their misconduct with the customers. Further, in case of any mishap or misbehaving, the registered taxi driver is punished with a low rating depending upon the behavior [7]. Further, the online cab services which provide cab booking facilities and customized pick up taxi convenience can be further automated and secured by connecting and analyzing using sensors. Every vehicle may connect to the IoT devices so that any malicious activity can be analyzed and averted. An intruder can try to alter the stored information or compromise the vehicle or IoT device for their selfish interests. However, using a secure mechanism, almost all activities can be traced in real time, such as traffic jams, weather conditions that hamper the drive, vehicular damage and repair etc. In addition, intruders may further compromise some IoT sensors in order to increase their credit points or to disable their location. Further, any alteration or change in stored data or information may not be transparently reflected to other drivers or users in the network that further encourages these people to continue their misbehavior [8]. As soon as the driver uses this application, his/her necessary information is registered somewhere and the vehicle is tracked through sensors during the nights for customer safety. Therefore, a blockchain technique has engrossed the attention of organization associates across a broad spectrum of industries, such as healthcare, real estates, transportation, government sectors and finance [9,10,11].The demand for transparency is rising at an astounding pace. In addition, it is able to ensure the security and transparency among the users in spite of IoT devices being vulnerable to intruders. Further, a blockchain technique is able to track, organize and bear out communications by storing the data from a large number of devices and facilitating the formation of parties without any federal cloud. The blockchain can confine the devices or CAV activities and trace the location from IoT objects when the cab moves from one place to another. Supply chain usage is the most relevant part of a blockchain technique for resolving the tangible harms to businesses due to the requirement of analysis of IoT devices or a vehicle’s legal or illegal activity information. Furthermore, the need of the blockchain in CAV is that it would capture the vehicle’s location, trace the vehicle’s position, record information or cab riding ratings phenomenon from IoT objects committed to the components or vehicle. Supply chain usage is the most general application of a blockchain for resolving real business issues due to the dearth of traceability of vehicle locations or in relation to the users or vehicles moving through the supply chain [12].

**Figure 1:**
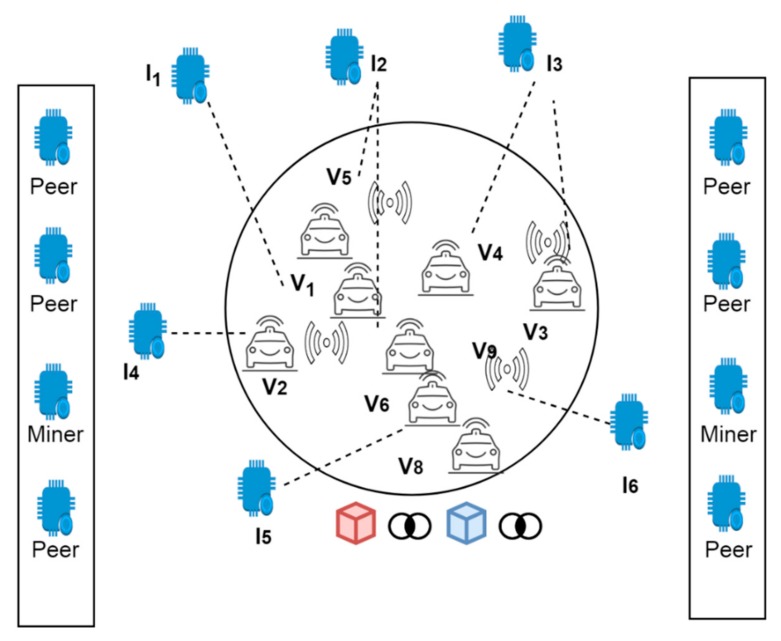
The blockchain framework.

Figure 1 depicts a typical vehicular blockchain network where IoT objects (I1, I2…I6) are connected among several peers. Further, the vehicles are considered as various peer nodes in the network which are further divided into miner nodes depending upon their service criteria. Miner nodes are responsible for validating the trust of remaining nodes or IoT devices while peer nodes are part of the entire blockchain.

### 1.1. Motivation of the Paper

The motivation of this manuscript is to provide security in the CAV network through IoT devices, safety and transparency among the users/customers during cab riding through a blockchain technique. During the movement of a user from one place to another, the vehicle number, current and previous vehicle ratings, captured through IoT devices are stored at the blockchain network. Therefore, even if intruders hack one or more IoT objects in order to gain their benefits, the users or vehicles present in the network are aware of the information registered under that compromised IoT device. Although, most of the researchers and scientists have proposed various smart frameworks in VANETs, however, most of the work is still at an early stage of CAV development. Further, many surveys have not affirmed the security concerns in the current progress of CAV applications.

### 1.2. Research Significance

Therefore, the scope of this paper is to ensure the transparency and security among drivers and customers using the blockchain. Further, the IoT sensors that automatically track the cab location and their identity using various IoT systems are traced by the blockchain network so that any compromise in any IoT device are recorded. The proposed blockchain framework has been validated against various security concerns such as user’s fake requests, the compromise of IoT devices, probabilistic authentication scenarios and alteration in stored users’ ratings. Further, the impact of using blockchain technology in IoV systems benefitted the society in variety of ways, such as ensuring the security and traceability of IoT devices or a vehicle’s legal or illegal activity information. The experimental analysis of the proposed framework has been measured upon the illegal activities or communications done by malevolent IoT objects. The percentage of vehicles data security upon compromising of IoT devices has been discussed.

The rest of the paper is organized as follows. The survey of literature related to VANETs, vehicles and the blockchain along with various applications is presented in Section 2. The proposed blockchain framework for online cab services is described in Section 3. Further, Section 4 analyzes the performance metrics of the proposed framework against certain networking scenarios. Finally, Section 5 clinches the work and highlights the future scope of the paper.

## 2. Related Work

Various authors and researchers have formerly presented reviews on several use cases of VANETs and the blockchain in different areas. However, very few researchers have introduced a blockchain technique in CAV. In this section, a detailed description of the CAV along with various security techniques and role of the blockchain in one of the VANETs known as IoV has been elaborated.

Lin et al. [13] have proposed an intelligent transportation system in order to assist the drivers for efficient traffic schemes, optimal routes and dynamic guidance of routes during their travel. In this paper, during real time scenarios for reducing the fuel consumption and travel time with less road congestion, the authors have proposed a dynamic en-route decision real-time route guidance scheme. Further, the author’s approach considers the real time traffic generation and transmission processes of the vehicular networks and predicts the traffic conditions based on multiple metrics by computing their trust probability. Furthermore, the authors have validated the proposed approach and shown the improved traffic efficiency by simulating the results in terms of efficient fuel, time and traffic parameters. Wang [14] introduced the basic methods and their major issues with their current applications for controlling and managing the traffic in parallel transportation management systems. The authors claim that the parallel transportation and management system is very effective for analyzing complex traffic networks. In this paper, the authors have described the parallel transportation and management system architecture, components, processes including Dyna, CAS, Itop, Adapts and Trans World. Finally, the author’s proposed framework has been experimented and validated by analyzing real-world applications.

Hamid et al. [15] illustrated the overview of IoV by explaining its emergence, history and current applications of IoV in autonomous vehicles. Further, the authors pointed out certain issues occurred during IoV connectivity with the environment and its security concerns. In order to verify the CAV importance, a case study with computational simulation is done. In addition, various research ideas and future work directions are listed in smart city highways. The authors omitted a detailed technical specification of CAV. Wu et al. [16] introduced the IoV with its various applications by describing the background, notion, the IoV network architecture and their characteristics analysis along with its new research and challenges. Further, the authors described some enabling technologies by illustrating MAC standards and routing protocols. In addition, the core of this paper is to present a complete taxonomy with several categories, such as efficiency services, driving safety, informative services and intelligent traffic management system. Finally, Wu discussed the future directions in IoV research. With the continuous increase in urban population and rapid expansion in cities, vehicle ownership has been increasing at an exponential rate. In order to keep this view, traffic management has become a great issue in day to day life of human beings. The motivation of Dandala et al [17] in this paper is to provide a traffic management solution using CAV for overcoming the issues prevailing in daily life. Further, Liang et al. [18] provided an overview of VANETs from a research viewpoint. The paper begins with describing the basic network architecture by discussing three popular research issues and general methods. At last, the authors end with the analysis on research challenges and future drifts. Rawat et al. [19] presented data falsification threat detection using hashes for improving the network performance and security by acclimatizing the contention window size to broadcast accurate information to neighboring vehicles in timely manner. Further, the authors have proposed a clustering scheme to overcome travel time during traffic jams. The proposed mechanism is validated through numerical results attained from virtual simulations. Qian et al. [20] added cognitive engines in traditional CAV by restricting security strategies and transmission delays. Further, the study specified the switches of path selection as 0-1 programming issues and non-convex optimization problems. In addition, the 0-1 programming problem is converted into non-convex optimization via a log-det heuristics algorithm. The proposed mechanism is validated through experimental results. Sharma [21] proposed an efficient model proficient of handling energy demands of the blockchain enabled IoV by optimally controlling the number of transactions through distributed clustering. The simulated numerical results suggest that the proposed approach is 40.16% better in terms of energy conservation and 82.06% better in terms of transactions required to share the entire blockchain data compared with the traditional blockchain. Castillo et al. [22] discussed the IoV benefits along with topical industry standards expanded to endorse its implementations. They further present proposed communication protocols to facilitate operation and seamless integration of CAV. At last, IoV future research work was presented by requiring further deliberation from the vehicular research community.

Furthermore, Pustisek et al. [23] briefly explained the blockchain technique by outline architecture in the automatic selection of an electric vehicle charging station. Several blockchain use cases for prototypic implementation were presented. Various security concerns exist due to high exposure of information and data flow between vehicles to intersection and vehicle to vehicle. Buzachis et al. [24] proposed a blockchain framework for verifying, negotiating and facilitating among the consent entities. In this paper, the authors proposed a multi agent vehicle to intersection and vice versa communication to secure the vehicles through intersections. Further, Kuzmin et al. [25] introduced the concept of blockchain in unnamed aerial vehicles where each vehicle is considered as a node in which the functionality for reading and creating the transactions or communication exchange in done through the blockchain network. Yang et al. [26] used the concept of blockchain during the sharing of traffic flow among vehicles by ensuring the tamper resistant and data correctness in the agreement mechanism. The proof-of-event agreement is used to collect the traffic data bypassing of roadside vehicles. A two-phase transaction is introduced to access the warnings through the blockchain. The simulated result validates the proposed mechanism against tracing the events with trust verification. Further, in order to ensure the vehicles security, the authors proposed various authentication mechanisms by restricting the several attacks. By identifying various attacks such as replay, location spoofing, guessing and authentication time requirement, Chen et al. [27] proposed an improved security mechanism by forming a formal proof. Furthermore, the proposed mechanism is validated by comparing with existing results in terms of performance and security. Moreover few researchers have focused on secure information exchange between various vehicles where any intruder has the capability to disrupt integrity, authenticity, confidentiality. In a smart city for ensuring a secure message exchange, Dua et al. [28] proposed a novel elliptic curve cryptographic mechanism for providing two level authentications. For validating the proposed mechanism, the analysis is done using burrows logic along with formal and informal analysis using internet security protocols. Further, the proposed mechanism is compared with existing security schemes against high reliability, latency and overheads.

Malicious users in CAV may mislead the whole communication and create chaos on the road. Further, data falsification attack is one of the main security issues in CAV where vehicles rely on information received from other vehicles or peers. Until now, the numbers of secure CAV mechanisms have been proposed by different researchers and scientists, however, very few works have presented CAV with a blockchain technique. This paper has proposed the issue of IoT sensors which are compromised by expert intruders by proposing a blockchain framework.

## 3. Proposed Blockchain Framework for CAV Services Delivery

This section describes the blockchain framework of CAV that ensures the security and transparency of users and vehicles. In order to trace each and every activity of malevolent resources, a security mechanism is proposed that keeps track of each activity done by IoT sensors. Therefore, for providing and ensuring the security during ride sharing in CAV, each transmission among entities through smart devices is tracked. Although it would be easy to trace or record each and every activity of vehicles, however it may further enhance the complexity of computational communication during tractability in real time scenarios where upon the mobility of vehicles, intruders may attack through denial of service or man-in-middle threats. Whereas, in a case where IoT devices keep record of each and every vehicle, any attempt by intruders to compromise the IoT devices can be easily traced and identified. Also, since IoT devices form an upper layer in the network, the probability of attack is significantly reduced as compared to edge level comprising vehicles. For ensuring the security of smart devices, registered providers are verified, so that nobody changes, alters or tracks the information or IoT devices after they are casted. Also, individuality in money bank vehicles is completed, so that nobody can steel any tracking information. These issues can be easily resolved by a blockchain technique where the required smart contracts are defined which is the same as writing the rules, models, objects and code among the parties. Smart contracts are considered as a consensus or an agreement between the two parties. Once the smart contracts are designed, they cannot be further deleted or altered from the blockchain network. In this mechanism, there is no need of a central authority to provide validation of the work. All the nodes or vehicles may compute their results of contracts without any outside interference.

**Figure 2:**
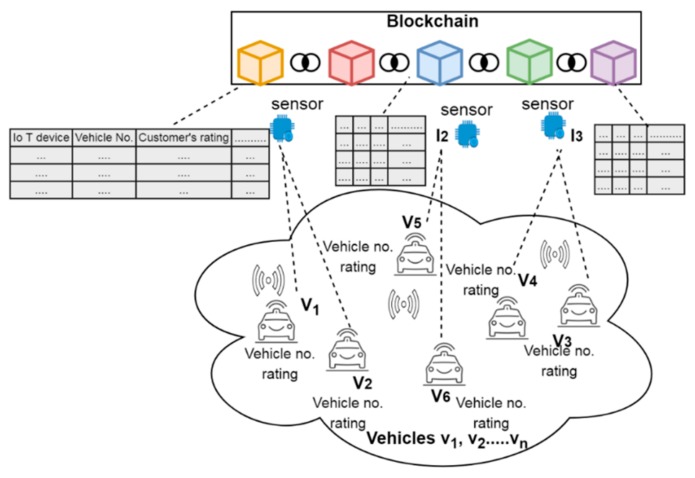
The architectural framework of a connected vehicle blockchain.

In the proposed blockchain framework, every automated vehicle or IoT device is registered or logged into the network before providing or accessing the vehicular services. Further, the necessary information of both vehicles and IoT devices are entered into an ordinary database initially and then stored in the blockchain permanently in order to track each and every activity of both the entities. Figure 2 depicts the architectural framework of CAV using the blockchain technique where all the vehicles are connected to IoT sensors or smart devices in order to control, monitor and guide the drivers on the road. In the proposed framework, the number of vehicles connected to the IoT devices or sensors depend upon their communication and transmission ranges. The vehicle number, ratings given by customers or users along with their IoT device are stored in ordinary tables as well as in the blockchain network to keep track and record each legal and illegal activity of the vehicle or IoT devices. In case of any IoT device being compromised by the intruders, the respective authorities which are part of the blockchain may be able to identify and take immediate actions against that compromised IoT device. Instead of recording IoT devices, each vehicle can be traced, analyzed and recorded over the blockchain. However, the keeping of records of such huge vehicular data during their mobility in real time scenarios may increase the possibility of computational power and time. Therefore, in order to limit the storage and computational power, it can be easy to record, analyze and store the activities of only IoT devices in the blockchain. The devices which trace a certain number of vehicles and provide services according to the user’s request can be easily traceable and recordable in the blockchain. Each IoT device containing its vehicle record and providing services to different vehicles can be used as mining information to store over the block. Any change or alteration in information communication in vehicles or devices by intruders can alter the history or previous interactions that may further punish the devices or vehicles by blocking or reducing the ratings of vehicles. This paper details the security of both vehicles and IoT device through the blockchain in two different cases. 

### 3.1. Vehicle Security: Registration of Every IoT Device on the Blockchain Network

**Figure 3:**
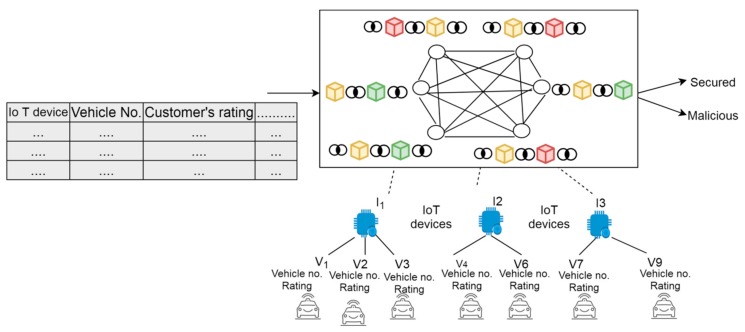
The blockchain network.

In order to ensure security and transparency during a ride, every IoT device that provides the information about the vehicles registers itself on the blockchain network before providing the services to the vehicles. Further, every vehicle number or rating given by the customers is stored on the blockchain network. In CAV, smart objects continuously monitor and control the cab services and each IoT device authenticates to a peer in the blockchain network as depicted in Figure 3.

The blockchain network is a combination of peer and miner nodes that are responsible for generating the cryptographic keys and verifying the authenticity of a new vehicle or IoT device joining the network in order to avoid network failures. More than one manager is elected to ensure the security in the network for a particular period of time. Once the manager is selected, a secondary blockchain manager is chosen in order to recover from the failure of the primary manager. All the IoT devices or vehicles (providers) are registered in the blockchain network, by sending a subscription request to the peer manager. Further, the authenticity or legitimacy of each IoT device or provider is verified by the miner nodes with the help of device information, such as the international device identity, device identifier, innovation technology crop etc. Once the authentication is successful, miners generate a shared key that will help in further validation. At last, all IoT devices connect with their assigned peer nodes using their shared key between peer nodes and IoT devices/objects.

**Figure 4:**
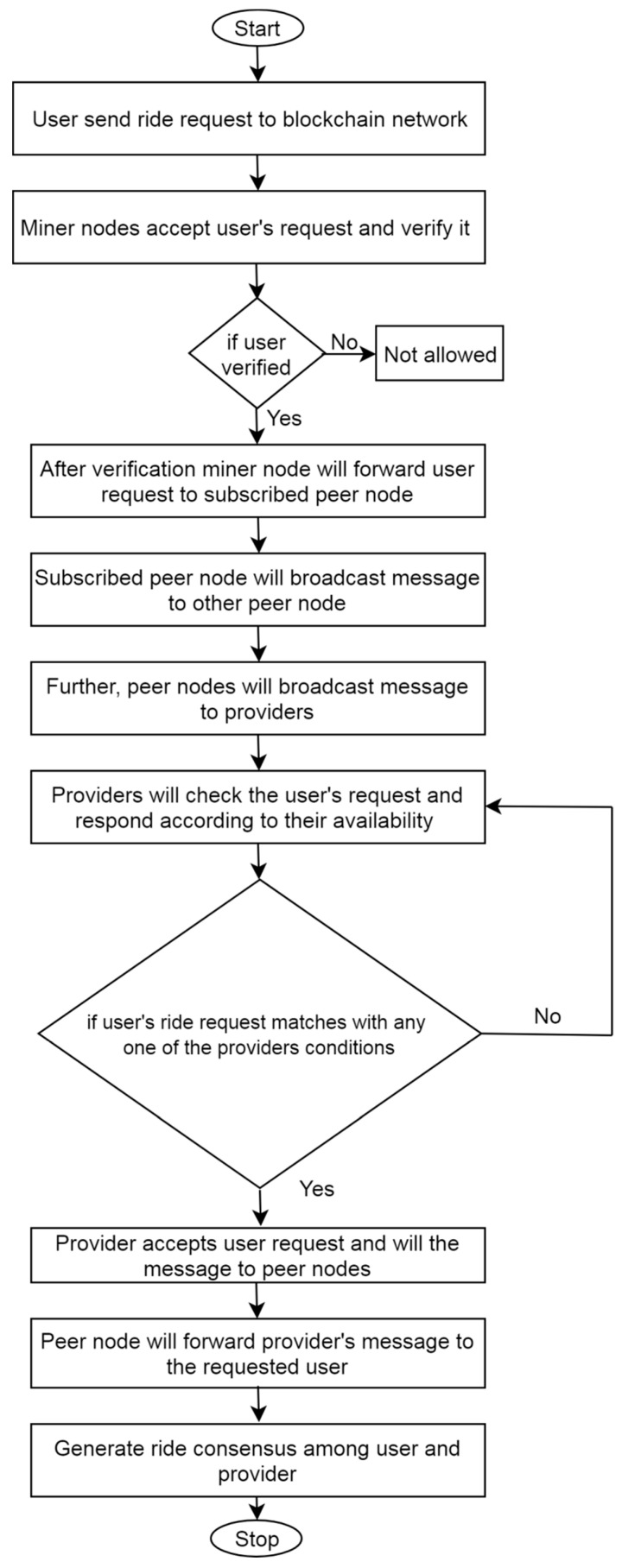
Flow work of the proposed framework.

Each vehicle acts as a node connected to its subscribed or nearby peer nodes. The flowchart of the proposed framework is depicted in Figure 4. As depicted in Figure 4, whenever a user X needs to book a ride, he makes a ride request by sharing the time, pickup and drop-off points of the ride. In this paper, the users or customers are considered as legitimate and need not submit their identity in the blockchain network. This ride request is visible to all the registered providers in the network who are the part of the blockchain. A rider may get positive or negative reviews from other users based on their behavior. Various parameters are used in order to compute the ranking of providers such as trust factor or rating. The provider with a high trust factor (TF) or rating is considered to be most trustworthy. The user may choose its provider depending upon the rating or TF. In the cab ride service, various communications are performed between the service provider and the requester. If provider Y wants to respond to this request, it can share its intent to X.

**Figure 5:**
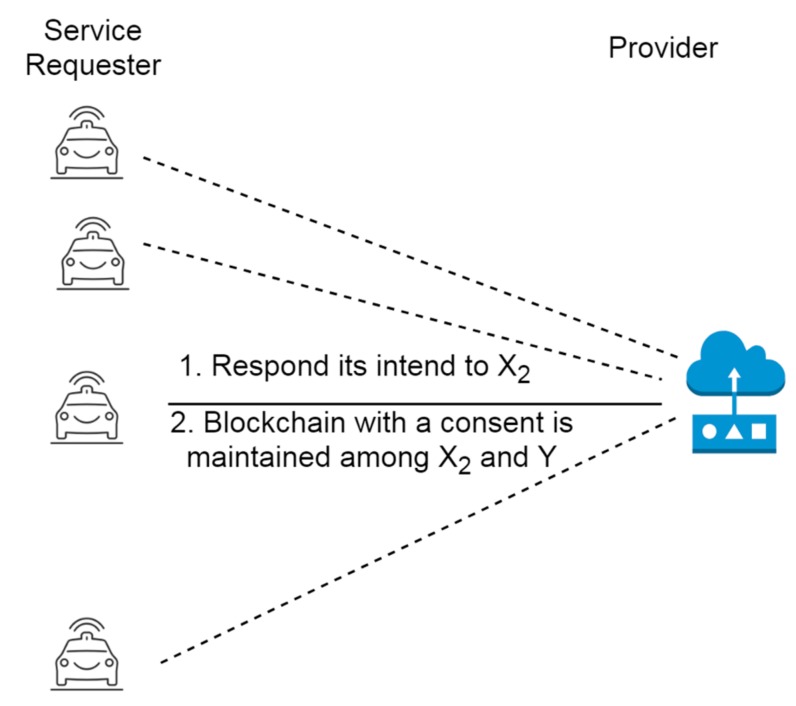
Consent through the blockchain among provider and ride requester.

**Figure 6:**
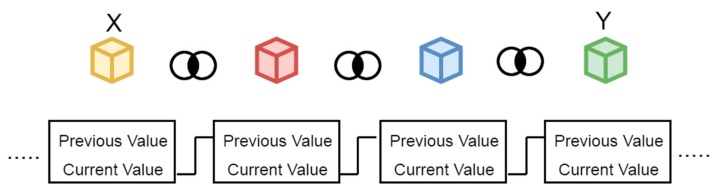
The blockchain among sender and receiver.

The provider Y chooses to respond to a ride request based on certain criteria such as the user’s route, where if the travel route of Y matches with the route of X, then Y accesses the ride request. Whenever a user X or provider Y’ agree upon a ride request, a blockchain can be maintained along with a hash so that any misbehavior or alteration in the location pick or drop point can subsequently be identified in the network as depicted in Figure 5. Each block contains the information about the IoT devices attached with a previous block through a hash as depicted in Figure 6 so that any alteration or deletion of any information from the intruder can come to the notice of other devices.

### 3.2. Attacking Scenarios

Whenever an intruder wants to perform some malicious activities in the network, it may adopt a number of attacking strategies. The compromise of IoT devices or sensors, modification of ratings given by riders, data falsification and traffic jams are some issues that can be easily generated by the intruders in order to fulfill their own interests. Attacking scenarios that can be possible during a ride service between user and vehicle are detailed as follows:

- Addition of compromised IoT by intruders: When the intruder registers its compromised IoT for executing its active or passive attacks, the blockchain peer nodes immediately identify by checking its illegal actions, like stealing or compromising of legitimate IoT devices.
- Misbehaving with the user: For example, a user, Alice, asks for a ride and a cab driver (provider) agrees to give the ride. However, during the ride the provider starts misbehaving with the user either by changing the route as chosen by the user, Alice or by stopping unnecessarily. Then, the IoT sensors which continuously monitor or trace the location of that cab takes action in order to prevent the user from any mishap. Simultaneously, the cab driver should be at the receiving end of the punishment with degradation in its rank or other necessary actions.
- Modification of ratings: Once the ratings have been submitted corresponding to any cab driver, it cannot be altered even after successfully compromising the IoT devices.
- Data falsification attack: It is one of the main security issues in CAV where vehicles rely on information received from other vehicles or peers.
- Traffic jam: In this, the intruders may try to divert the path suggestions on the roads for their own benefits.

However, in order to prevent these attacking strategies, this study has proposed a secure cab riding and sharing mechanism through the blockchain. Further, in order to validate the proposed mechanism, a numerical simulation is done on various parameters that show the outperformance of the proposed framework. 

## 4. Performance Analysis

**Figure 7:**
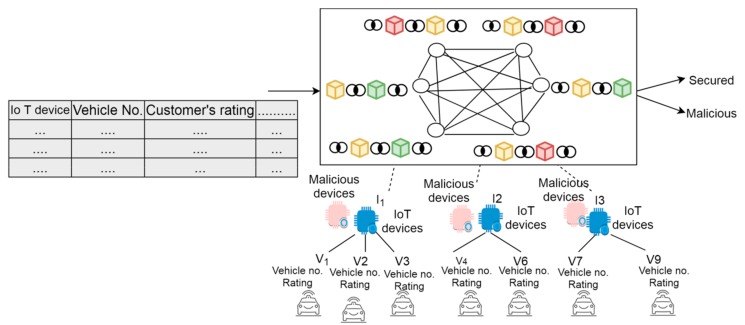
An adversary network model of the proposed phenomenon.

For validating the proposed framework, the simulation of the CAV blockchain framework has been ensured using the blockchain technique through NS2 simulator. In this paper, the possibility of attacks encountered at IoT devices or vehicles of the proposed framework has been analyzed. Initially, a network area of 700 × 700m was created having network sizes of 50 numbers of nodes where the vehicles are dynamic in nature and can abscond and join any other device’s range as depicted in Table 1. For the deployment of network establishment, an initial random rating or TF (such as 70% and 5) was also assigned on the network to each device/vehicles and 5 nodes were created that act as blockchain nodes. In order to measure the validity of the proposed phenomenon, the performance was measured against several security metrics, such as the user’s fake message alteration, the attack possibility on IoT devices and the modification of users’ record information, such as the ratings. In order to measure validity or verify the proposed blockchain’s CAV framework, NS2 simulator was used where numbers of attacking scenarios at IoT devices were considered. An attacking scenario or adversary model of the proposed framework is depicted in Figure 7 where intruders compromise the IoT devices either by forging the identity of legitimate devices or hacking the existing legitimate IoT sensors in the network. In order to validate or measure the authenticity of the proposed phenomenon, the attacking nodes were added with the rate of 10% for legitimate nodes in the network. 

The intruders that try to compromise the IoT devices either by hacking the legitimate devices’ identity (ID) in order to perform man-in-middle attack or behave like legitimate devices are considered for analyzing the proposed framework. Further, the proposed phenomenon has been verified against network congestion and compromising ability where intruders consume network resources by broadcasting fake requests. In addition, the proposed framework was measured against authentication probabilistic scenarios where attackers try to compromise IoT devices and showed how the possibility of attacks can still be analyzed and measured where intruders compromise the IoT devices. The proposed phenomenon showed false, no and correct authentication scenarios values that depicted how the proposed phenomenon efficiently measured the attacks where intruders compromised the devices. Further, in order to verify the framework against threats, malicious devices or nodes were added into the network based on normal distribution during the communication process. Further, the established blockchain environment is a combination of miners and peer nodes for validating and adding the new nodes (devices) in the network. Amongst them, some miner nodes were also converted to malevolent nodes to see the security recovery process. In addition, IoT devices were considered which are under the threat of intruders. The invasion of IoT signified that in a single unit of time, 2 out of 5, 10 out of 20 and 20 out of 50 devices were compromised as depicted in Table 2. Further, the user’s fake request was considered another threat where insertion of a fake request by the intruders caused network congestion in the network. Taking all these assumptions, performance analysis was done for 60 s. The proposed framework was validated and compared against conventional approach as discussed in the below subsection.

### Existing Method

Rawat et al. [17] presented data falsification threat detection using hashes for improving the network performance and security by acclimatizing contention window size to broadcast accurate information to neighboring vehicles in a timely manner. Further, the authors proposed a clustering scheme to overcome travel time during traffic jams. The existing mechanism was validated through numerical results attained from virtual simulation. The proposed paper analyzes the blockchain mechanism of IoT framework for CAVs over various networking parameters, such as the users’ fake requests, compromise of IoT devices and alteration in the stored user’s ratings. The proposed framework was measured against Rawat et al. [17] where the authors ensured the security by generating the hashes of information transmitted among the entities. However, the encrypted messages can be easily hacked and altered by the intruders. Further, a single change or compromise of IoT devices in the CAV network may be unaware of the entire network. However, in our proposed mechanism a single change in any information or device may immediately alert the remaining networks.

The proposed phenomenon results are measured against two attacking scenarios, i.e. network congestion and compromising ability that is further compared against existing approaches explained in subsection A of Section 4. The proposed method tries to improve the security of vehicles through the blockchain where every IoT device is recorded and analyzed for detecting the threat possibility. Rawat et al. on the other hand proposed a phenomenon where a block of hash chains of each vehicle was recorded over the network that may further have enhanced the possibility of an attack due to network congestion and computational power at the lower level of vehicles. The experimental evaluation of the proposed and conventional approaches was accomplished successfully and multiple results regarding various parameters have been recorded. The performance and system state parameters results are presented in previous subsections of the performance analysis. The system behaved as expected and all performance parameters for any CAV data were positive for the proposed framework. The movement and recording of activity is done by IoT devices that are static and can analyze and detect efficiently. The movement of vehicles allows new connection to the IoT device of their range where devices may collaborate among each other to further analyze their interactions.

**Figure 8:**
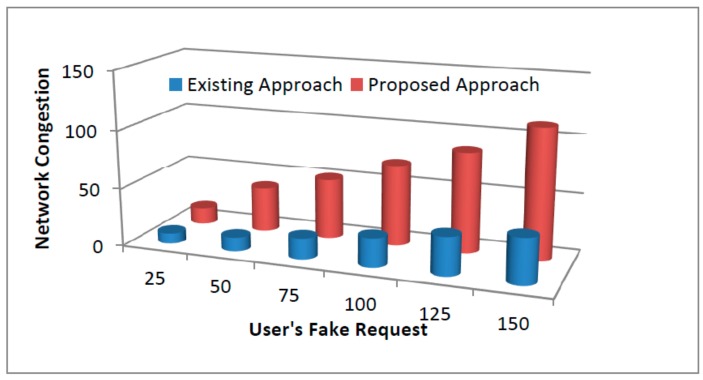
The users’ fake requests corresponding to network congestion.

**Figure 9:**
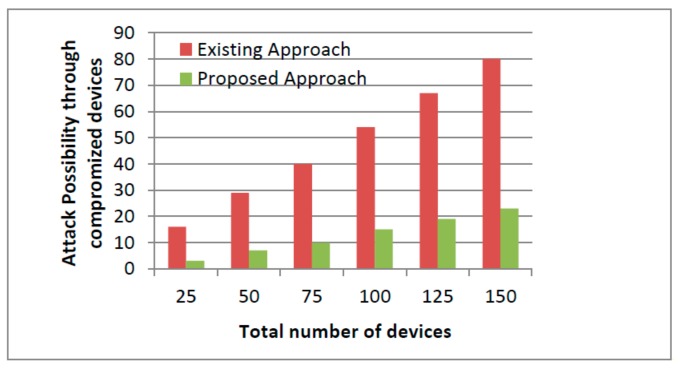
The attack possibility against compromised devices.

**Figure 10:**
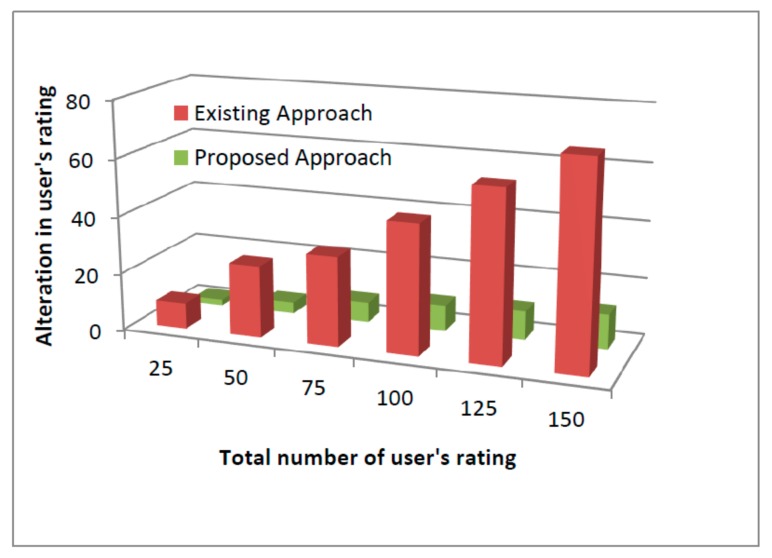
In user’s stored ratings by intruders.

Further, the accuracy of the proposed approach was close to 86% which may improve with the time because of removal of detected malicious nodes (MNs) from the system. The detection of MNs is based on trust where removal of detected MNs does not hinder the performance of other nodes. The proposed mechanism computes the trust of other nodes after every specific interval of time where nodes that are compromised and behave maliciously can have lower rating and trust because of high product loss ratios, black holes, and falsification attacks and may never be considered in the future. The depicted results showed the outperformance of the proposed mechanism against existing approaches with a success rate of 86%. This can be further improved if the experiment runs for a longer period. The measuring parameters in the proposed framework performed better in comparison to existing systems. Further, the obtained framework accuracy can be further improved with time because of the removal of detected MNs from the network. The detection of MNs followed by their removal does not alter the trust or hinder the performance of other nodes. The proposed mechanism computes the trust factor of their nodes after a specific interval of time. The nodes that are compromised and behave maliciously can have low ratings and trust, and may never be considered for the path formation. In all the depicted graphs from Figure 8, Figure 9 and Figure 10, the proposed security framework outperforms better results against existing mechanisms. In case of the user’s fake request graph as shown in Figure 8 corresponding to network congestion, the existing scheme performs less efficiently as the number of fake requests increase with the network size. The congestion of fake requests overloads the network and communication between the sender and the receiver and can become very difficult to maintain. Further, the increase of network congestion may consume the necessary resources that further leads to drastic degradation in network performance. Furthermore, corresponding to compromised devices, the data monitoring and controlling mechanism was affected highly as shown in Figure 9. In case of compromised IoT devices, the intruders not only affect the network performance, but also gain access to restricted areas or may further steal the confidential information for their own benefits. However, in the case of Figure 10, the intruders may alter the stored ratings of users and continue their misbehavior with their customers.

**Figure 11:**
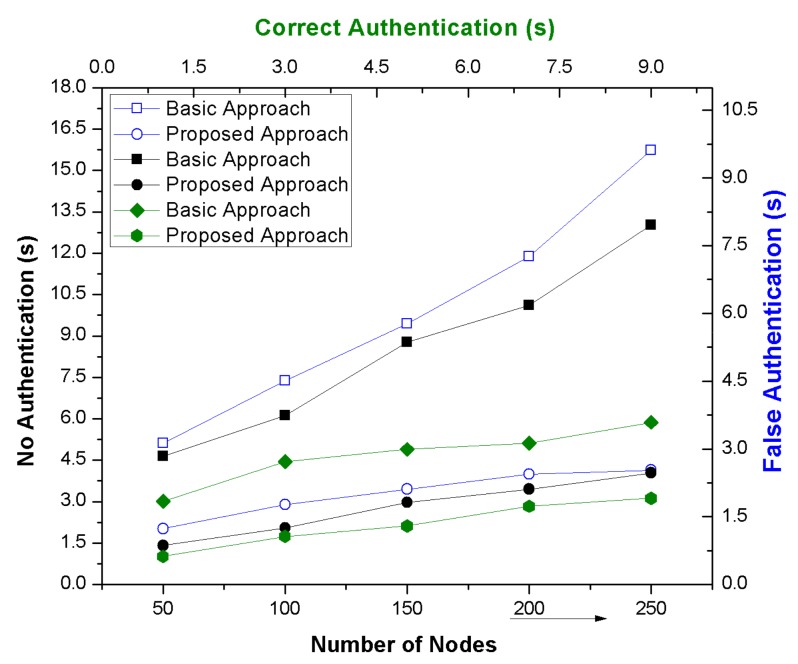
Probabilistic scenarios of attack possibility.

Along with the blockchain technology, all the necessary documenting or monitoring or controlling records are stored at the blockchain so that a single alteration, modification, deletion or compromise of any IoT device may quickly get under the surveillance and be known to other devices in order to secure or prevent from future possible harms. In our proposed phenomenon, a vehicular security framework was projected with the blockchain technique that enhanced the network performance and secured the online cab services. The performance analysis of proposed framework was further explained in detail along with verification time depending upon its various probabilistic attacking strategies. Figure 11 depicts the probabilistic strategies in terms of false, no and correct authentication and illustrates the validation of the proposed phenomenon with fake requests and compromising ability. The approach can be applied efficiently in real time scenarios by measuring its attacking possibilities.

## 5. Conclusions

This paper has considered the IoV application and proposed a security mechanism for connected autonomous vehicles services framework using the blockchain technique. In order to provide the secrecy and transparency among the customers and cab drivers, each activity of the entities regarding vehicles or IoT devices is traced and recorded inside the blockchain. The blockchain mechanism is used to extract the information from IoT devices and store the extracted records in order to ensure the customer’s safety and the devices’ security by providing transparency among various authorities. The proposed framework significantly reduced the users’ fake requests, the compromise of IoT devices and the alteration in the stored user’s ratings. The simulated results against various parameters showed a 79% success rate in the proposed framework as compared to the existing approach against mentioned parameters. The proposed phenomenon against a larger number of nodes and the transaction alteration already stored at the blockchain network will be reported in future communications. Further, technology such as deep and reinforcement learning will be adopted to increase the system intelligent [29].

## Figures and Tables

**Table 1:** Parameters.

Number of Nodes in a CRN	25, 500
Grid facet	700 × 700 m
Transmission Range	140 m (approx.)
Data Size or users request	256 Bytes
Simulation time	80 s
Physical Layer	PHY 802.11

**Table 2:** The configuration of NS2 for a different network environment.

S. No.	Transmitting Nodes	IoT Nodes	Compromised Miners	Attack Probability
1	25	5, 10, 20	2, 10, 20	5%
2	100	25, 50, 75	15, 25, 50	25%
